# Case report: Sphenoid wing dural arteriovenous fistula draining into ophthalmic veins inducing pulsatile tinnitus as the sole symptom and its spontaneous closure

**DOI:** 10.3389/fneur.2023.1293899

**Published:** 2024-01-11

**Authors:** Yue-Lin Hsieh, Jiake Zhong, Xi Chen, Wuqing Wang

**Affiliations:** ^1^ENT Institute and Department of Otorhinolaryngology, Eye & ENT Hospital, Fudan University, Shanghai, China; ^2^Department of Radiology, Huashan Hospital, Fudan University, Shanghai, China

**Keywords:** pulsatile tinnitus, sphenoid wing, dural arteriovenous fistula, superior ophthalmic vein, spontaneous closure

## Abstract

This case report discusses a unique instance of pulsatile tinnitus (PT) caused by a rare type of intracranial dural arteriovenous fistula (DAVF) located in the sphenoid wing (SW) region, with PT being the sole presenting symptom. The patient initially received multiple misdiagnoses and sought medical attention at various hospitals before being correctly diagnosed. Imaging studies revealed the DAVF’s presence in the SW region, which led to the patient’s referral to interventional radiology/neurology, although she chose conservative observation without surgical intervention. Remarkably, the patient’s PT spontaneously ceased after 30 months without any apparent cause, and follow-up imaging confirmed the absence of DAVF-related abnormalities. The case highlights the importance of considering DAVF as a potential cause of PT, even when there are no evident abnormalities in proximity to the auditory apparatus. It also emphasizes the need for otolaryngologists to extend their examination to include regions beyond the temporal bone, such as the sphenoid bone and orbital areas, when PT is the exclusive symptom. The case underscores the significance of early detection and intervention for DAVFs, as they can lead to debilitating complications, despite the rare occurrence of spontaneous symptom resolution in this case.

## Introduction

Vascular pulsatile tinnitus (PT) arises from mechanical sounds induced by blood flow, frequently occurring in proximity to the temporal bone, where osseous and vascular anomalies are prevalent (1, 2). Typically, it originates from heightened flow kinetic energy resulting from vascular irregularities or the transmission of sound waves through defects in the temporal bone, with sigmoid sinus wall anomalies being the most frequent culprits (3). Conversely, intracranial dural arteriovenous fistulas (DAVFs) have also been documented as a common source of PT, often accompanied by other concurrent neurological symptoms (4). In contrast to venous etiologies, DAVFs demand immediate attention due to potential complications, including hemorrhage, progressive symptoms, venous thrombosis, or cortical venous drainage (5).

A DAVF is an unusual connection between arteries and veins within the dura mater, the outermost membrane enveloping the brain or spinal cord, accounting for 10 ~ 15% of all intracranial arteriovenous malformations (6). A DAVF located at the sphenoid wing (SW), which represents approximately 1% of all intracranial DAVFs and drains into the superior ophthalmic vein (SOV), is a rare subtype among intracranial DAVFs (7). SW-SOV DAVFs primarily manifest with ocular symptoms, including visual disturbances, proptosis, and chemosis (7–10).

While up to 70% of transverse-sigmoid sinus DAVFs have been reported to have PT, PT as a standalone symptom in DAVF patients comprises only approximately 10% (4). Notably, PT has been reported in 35.7 ~ 41% of SW-SOV-associated DAVFs, most of which are often accompanied by proptosis, chemosis, and other neurologic deficits (7–10). To date, there have been no reports of PT as the sole symptom of SW-SOV DAVFs.

This study presents a unique case of Cognard IIa SW-SOV DAVF with PT as the sole symptom and a rare incidence of the spontaneous closure of the fistula. The absence of ocular symptoms can lead to the radiologic oversight of a relatively small and inconspicuous SW-SOV DAVF in the medial middle cranial fossa. This case underscores the importance and educational value for otolaryngologists to thoroughly examine the orbital region, in addition to the temporal region, when managing DAVFs associated with PT as the only presenting symptom.

## Case presentation

A 31-year-old female patient, who had been experiencing left-sided PT for the past 10 months, sought our clinic’s assistance in November 2020. She had previously received multiple misdiagnoses, including migraine and subjective tinnitus, and had sought medical attention from at least three local tertiary hospitals in the ENT department. Her PT persisted despite attempts at reducing it through internal jugular vein compression. Nevertheless, her PT showed a slight decrease in volume following the application of strong compression to the internal carotid artery using a Doppler ultrasound probe. PT could not be discerned through transcanal recording or stethoscope auscultation. Ocular assessments, such as fundoscopy, visual field examination, and orbital ultrasound, uncovered no irregularities. A physical examination did not reveal any signs of heightened conjunctival vascularity, exophthalmia, oculomotor paralysis, or visual acuity loss. Her medical history is unremarkable, and she had no previous trauma.

**Figure 1:**
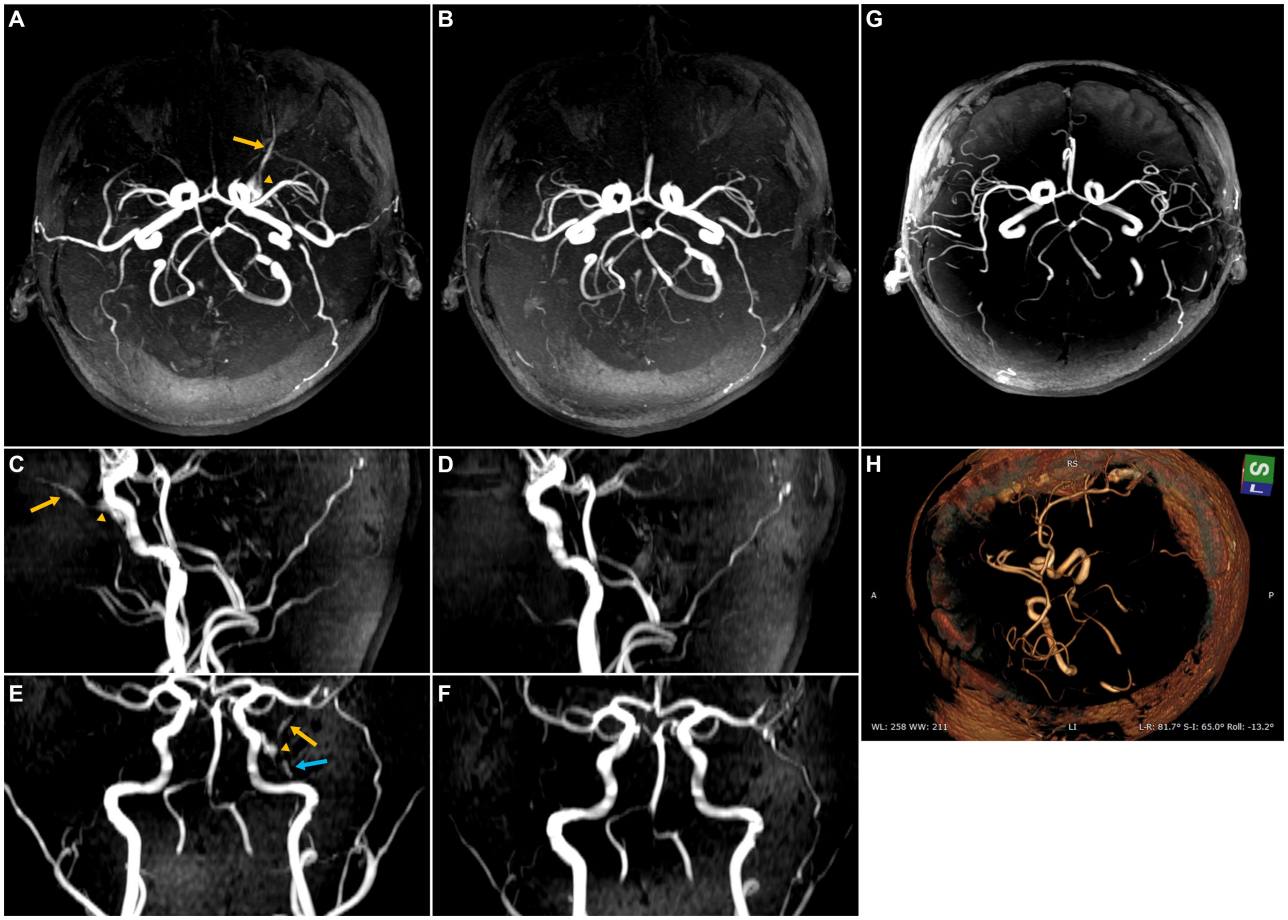
Maximum intensity projection (MIP) images from 3D time-of-flight (TOF) magnetic resonance (MR) angiography illustrating the sphenoid wing (SW) dural arteriovenous fistula (DAVF) both before and after self-healing. The yellow arrow highlights the SOV primary efferent pathway of the SW DAVF, while the yellow triangle indicates the para-cavernous sinus (para-CS) drainage, and the blue arrow suggests the possible involvement of the efferent inferior ophthalmic vein. **(A)** Axial MIP image before self-healing. **(B)** Axial MIP image after spontaneous closure, possibly attributed to thrombus embolization. **(C)** Sagittal MIP image before spontaneous closure. **(D)** Sagittal MIP image after self-healing. **(E)** Coronal MIP image before spontaneous closure. **(F)** Coronal MIP image after spontaneous closure. **(G)** Follow-up MR angiography was performed 42 months after the initial onset of PT, depicting the absence of radiologic indications of DAVF. **(H)** Volume rendering technique was applied to the 42-month follow-up MR angiography, showing the absence of radiologic indications of DAVF.

**Figure 2:**
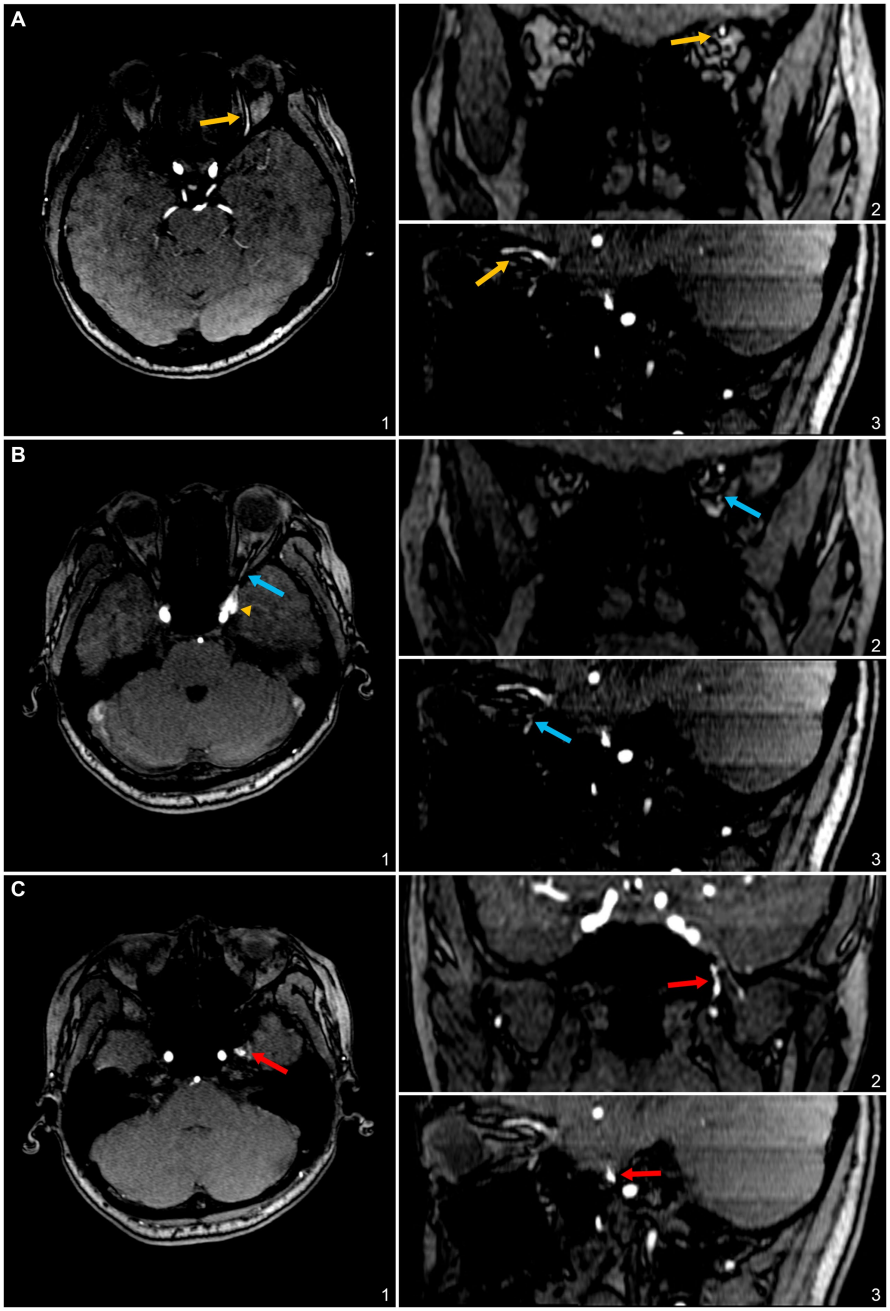
3D time-of-flight (TOF) magnetic resonance (MR) angiography slices highlighting the involved efferent venous pathways of the sphenoid wing (SW)-DAVF in this case. **(A)** Axial (1), coronal (2), and sagittal (3) slices depict the superior ophthalmic vein (SOV) as an efferent pathway, denoted by yellow arrows. **(B)** Axial (1), coronal (2), and sagittal (3) slices illustrate the inferior ophthalmic vein as a potential efferent pathway, indicated by blue arrows. **(C)** Axial (1), coronal (2), and sagittal (3) slices display the pterygoid plexus, marked with red arrows.

The patient had undergone both computed tomography (CT) and magnetic resonance (MR) scans. CT showed no signs of temporal bone dehiscence at either the sigmoid plate or the jugular bulb regions. The three-dimensional time-of-flight (TOF) MR angiography showed a relatively small region of hyperintense signal at the lesser SW region and the left para-CS (laterocavernous sinus) region (Figure 1), which extends to its efferent outlets, typically the SOV and inferior ophthalmic vein (IOV), and through the meningeal emissary veins to the pterygoid plexus (Figure 2). Upon this discovery, the subject was referred to interventional radiology/neurology. However, due to the patient’s unwillingness to undergo surgical intervention or digital subtraction angiography (DSA) examination, the subject chose conservative observation and took no medication, but was kept under follow-up care.

## Results

The subject’s PT abruptly ceased without any apparent provocation after persisting for a total duration of 30 months. The tinnitus handicap inventory score decreased from 32 to 0. A subsequent 3D-TOF MR angiography follow-up examination failed to detect any residual high-intensity signals within the SW region, para-CS, or any of the associated efferent vessels. Furthermore, the subject did not report experiencing any ocular symptoms or adverse effects subsequent to the cessation of PT. A follow-up MR angiography was conducted 42 months after the initial PT onset, revealing no signs of PT recurrence and no radiologic indications of DAVF (Figure 1).

## Discussion

This case study serves to underscore the occurrence of PT associated with DAVF in the SW and orbital regions, even in the absence of discernible hyperintense signals on MR angiogram screenings focusing on pathologies proximate to the auditory apparatus. Consequently, it is imperative for otolaryngologists to extend their examination to encompass regions beyond the temporal bone, including the sphenoid bone and orbital areas, notwithstanding PT being the exclusive presenting symptom.

DAVFs in the lesser SW region are often characterized as anomalous connections between the middle meningeal artery and the sphenoparietal sinus (7). Even in the absence of DSA confirmation, there exists a significant likelihood that the fistula site, in our case, involves the left sphenoparietal sinus, with drainage into the para-CS region, SOV, IOV, and the pterygoid plexus. In cases where multiple efferent venous pathways are adequately functioning, we infer that our subject is consequently devoid of ophthalmic and neurological complications other than PT.

In the context of PT pathophysiology, it is plausible that PT originates from the generation of flow-induced sound waves or vibrations in adjacent tissues, precipitated by the abnormal redirection of high-pressure meningeal arterial branches into the low-pressure para-CS and venous efferent vessels. The produced sound/vibration propagates through the bone and solid tissue conduction route to reach the inner ear, rather than via the air-conduction pathway. This elucidates the inability to detect PT through trans-external canal recording.

Beyond PT being the sole presenting symptom, this case also signifies a unique instance of spontaneous symptom resolution induced by the Cognard IIa SW-SOV DAVF. Unlike larger SW DAVFs, sphenoparietal sinus DAVFs, which are located in the lesser wing of the sphenoid, usually have a milder clinical course. This is because they often have significant epidural venous drainage into the cavernous sinus, which lowers the chances of cortical venous reflux (8). As a result, the risk of intracranial venous hypertension, hemorrhage, and severe symptomatic presentation is reduced.

In a recent systematic review of 54 spontaneous regression cases in the literature, the cases documenting the spontaneous regression of intracranial DAVFs were almost evenly distributed between low-risk lesions, comprising 57.14% classified as Borden I and 44.82% categorized as Cognard IIa or lower, and aggressive high-risk lesions, consisting of 42.58% categorized as Borden II or greater and 55.18% classified as Cognard IIb or greater (11, 12). This lends support to the notion that lower-risk DAVFs do not necessarily indicate higher rates of spontaneous regression (12). Sinus thrombosis is recognized as a significant factor contributing to the spontaneous closure of DAVF. However, alternative mechanisms, such as intracerebral hemorrhage resulting in mass effect and secondary vasospasm, have also been proposed as potential explanations for the spontaneous closure of DAVFs (11). The resolution of DAVF can transpire via either the direct closure of the shunt or an enlargement of the sinus due to structural alterations, subsequently leading to the compression of the arteriovenous shunts within the wall, resulting in DAVF occlusion (12). It is worth noting that the second scenario, however, was not observed in our specific case. Therefore, we conjectured that the remission of DAVF and PT in this case is likely attributed to thrombus embolization leading to a spontaneous closure.

Nonetheless, it is of paramount importance to underscore that DAVF necessitates immediate medical attention, typically referring to an interventional radiology or neurology department, given that induced PT and the complications associated with SW-SOV DAVF can be profoundly debilitating, potentially culminating in complications such as venous thrombosis/infarction, intraparenchymal bleeding, and subarachnoid hemorrhage in some cases.

The limitation of this study is the absence of dynamic MR and DSA techniques employed to thoroughly examine hemodynamics, thereby limiting a comprehensive analysis of vascular dynamics and potentially impacting the depth of our understanding.

## Conclusion

DAVF, situated within the SW region, where the primary efferent vessel is the SOV, has the capacity to trigger PT as the sole symptom. In our case, SW-SOV DAVF can be easily overlooked if exclusive focus is placed on the transverse-sigmoid sinus or temporal bone alone. Remarkably, this case also constitutes the first reported instance in the literature regarding SW-SOV DAVF of the spontaneous remission of this condition.

## Data availability statement

The original contributions presented in the study are included in the article/supplementary material, further inquiries can be directed to the corresponding author.

## Ethics statement

The studies involving humans were reviewed and approved by the Ethics Committee of Eye & ENT Hospital of Fudan University, China. The studies were conducted in accordance with the local legislation and institutional requirements. Written informed consent was obtained from the individual for participation in this study and publication of any potentially identifiable images or data included in this article.

## Author contributions

Y-LH: Conceptualization, Data curation, Formal analysis, Investigation, Methodology, Validation, Visualization, Writing – original draft, Writing – review & editing. JZ: Investigation, Writing – review & editing. XC: Conceptualization, Validation, Visualization, Writing – review & editing, Formal analysis, Investigation, Writing – original draft. WW: Conceptualization, Funding acquisition, Resources, Supervision, Validation, Visualization, Writing – review & editing.
